# The role of NAFLD in cardiometabolic disease: an update

**DOI:** 10.12688/f1000research.12028.1

**Published:** 2018-02-09

**Authors:** Sarah Faasse, Hayley Braun, Miriam Vos

**Affiliations:** 1 Health Sciences Research Building, Emory University, 1760 Haygood Drive, W-450, Atlanta, GA 30322, USA

**Keywords:** non-alcoholic fatty liver disease, cardiometabolic disease, type 2 diabetes mellitus

## Abstract

Non-alcoholic fatty liver disease (NAFLD) is the most common cause of chronic liver disease in the world, yet the complex pathogenesis remains to be fully elucidated. The prevalence of NAFLD has risen precipitously in recent years and is now a leading indication for liver transplantation. New waitlist registrants with non-alcoholic steatohepatitis–induced cirrhosis increased by 170% from 2004 to 2013. In addition, patients with NAFLD are at increased risk of both cardiovascular disease and type II diabetes. In this update, recent studies contributing to the understanding of the place of NAFLD in cardiometabolic disease will be discussed.

## Introduction

Non-alcoholic fatty liver disease (NAFLD), now the most common liver disease in the world, is defined as the accumulation of fat in the liver beyond 5% of total liver weight in the absence of excessive alcohol use and other pathogenic sources such as viral hepatitis, Wilson disease, alpha-1-antitrypsin deficiency, autoimmune hepatitis, or other metabolic diseases ^1– 4^. The estimated prevalence of NAFLD in the US is now 19%, and worldwide prevalence estimates are between 25.24 and 32.5% ^5, 6^. The marked increase in NAFLD prevalence has occurred concomitantly with increases in rates of obesity and the metabolic syndrome (MS) worldwide. Obesity itself is heterogenic, and a greater understanding of the relevance of body fat distribution patterns to NAFLD has developed recently as a growing number of studies using magnetic resonance imaging (MRI) to measure fat distribution have been published ^7– 9^. The previous mainstay was dual-energy x-ray absorptiometry, useful for visceral fat ^10^, muscle mass, and total adiposity, but it misses hepatic fat. Body mass index (BMI) can both underestimate disease, as in the case of metabolically obese, normal-weight individuals ^11^, and overestimate disease, as in the case of obese (BMI of more than 30 kg/m ^2^) but metabolically healthy people.

People in the metabolically unhealthy category, regardless of BMI, tend to have increased visceral adipose tissue (VAT) ^12^. VAT is metabolically active and has been thought to be complicit in the dysmetabolic syndrome ^13^. Individuals with a higher proportion of VAT are more likely to have NAFLD ^14^ and a higher risk of associated dysmetabolic disease ^15, 16^. This leads to questions of which drives which: hepatic fat leading to VAT, or vice versa? There are two studies that demonstrate in both adults and adolescents that intrahepatic fat, rather than VAT, is most closely associated with insulin resistance (IR), suggesting that intrahepatic fat is the primary driver of metabolic dysfunction in the centrally located adiposity phenotype ^7, 8^. Within the liver, *de novo* lipogenesis (DNL) is the primary dysfunction of lipid metabolism leading to steatosis accumulation. In healthy metabolism, DNL is not a major contributor of lipids in the liver. However, in NAFLD, DNL is abnormally upregulated ^17^, and it increases further in the setting of weight gain ^18^. In NAFLD, approximately one fourth of the fat in the liver is directly from DNL ^19^.

Amino acid metabolism is another important area of disturbance in NAFLD, and newer techniques such as high-throughput, high-resolution metabolomics have advanced studies in this area. Specifically, levels of branched chain amino acids (leucine, isoleucine, and valine) and aromatic amino acids (tyrosine) have been found to be altered in NAFLD ^20, 21^. Notably, a similar amino acid profile was also found to predict risk of future type 2 diabetes mellitus (T2DM) ^22^. In pediatric patients with NAFLD, tyrosine metabolism was found to be most significantly altered in adolescent patients, and a recent study shows that elevated levels of tyrosine, glutamate, and glutamate/(serine+glycine) ratio (that is, GSG index) were associated with IR and that alanine, glutamate, isoleucine, and valine were increased in non-obese patients with NAFLD ^21, 23^. More studies are needed to understand whether dysregulated amino acids represent promising biomarkers or future therapeutic targets for NAFLD.

## Genetic factors

As is typical of many chronic diseases, NAFLD is a genetic condition with an environmental trigger that induces the expression of the phenotype. In the case of NAFLD, this trigger appears to be, at a minimum, a hypercaloric condition. The most established genotype complicit in liver steatosis is a mutation of the rs738409 allele of patatin-like phospholipase domain ( *PNPLA3*) ^24^. The mutation is a single-nucleotide polymorphism (SNP) of codon 148, resulting in a missense mutation of isoleucine to methionine (I148M) ^25^. The homozygous variant is most commonly found in Hispanics. A recent article found that adiposity potentiates the effect of this missense mutation, resulting in a higher risk for development of NAFLD as BMI increases in patients with this particular variant ^26^. Interestingly, another variant allele of PNPLA3 (rs6006460) that results in a serine-to-isoleucine (S453I) missense appears to have a hepatoprotective effect, associated with lower intrahepatic fat content, and is most commonly found in African-Americans, an ethnic group that has a relatively low prevalence of NAFLD ^27^. Other SNPs have been identified in the pathogenesis of NAFLD, glucokinase regulatory protein ( *GCKR*) rs1260326 ^28^, and transmembrane 6 superfamily member 2 ( *TM6SF2)* **rs58542926 ^29^, and when assessed with PNPLA3 rs6006460, these three SNPs seem to have an additive effect on determining intrahepatic fat content ^29^.

## Prenatal exposure

**Figure 1.**
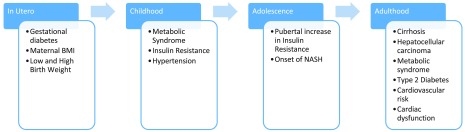
Temporality of risk factors, comorbidities, and potential consequences of non-alcoholic fatty liver disease. These findings suggest a common exposure or complementary or additive relationship between non-alcoholic fatty liver disease and other metabolic diseases. BMI, body mass index; NASH, non-alcoholic steatohepatitis.

It is vital to consider NAFLD within the life span of cardiometabolic disease as it is not an isolated disease ( Figure 1) ^30^. Some important studies have emerged that suggest prenatal origins for NAFLD. Friedman *et al*. conducted an elegant set of experiments in rhesus monkeys and demonstrated that a high-fat diet in insulin-resistant pregnant monkeys predicted fatty liver in the offspring, regardless of the diet (healthy or not) given to the infant monkeys ^31, 32^. Limited but intriguing evidence in humans supports this finding. Two recent MRI-based studies found that higher maternal BMI positively correlates with both higher postnatal infant adipose tissue and intrahepatic fat content, suggesting that the burden of intrahepatic fat begins *in utero* ^33, 34^. Similarly, infants born to obese mothers with gestational diabetes (GDM) had, on average, 68% more hepatic fat content by MRI quantification than those born to normal-weight women ^34^. An autopsy study of stillborn babies showed that histopathologic hepatic steatosis was significantly more prevalent in children whose mothers had maternal GDM (79%) compared with controls (17%) ( *P* <0.001) ^35^. Together, these studies suggest that maternal IR may program the liver during development and drive hepatocyte function toward steatosis. More information is needed regarding maternal environmental exposures and the effect on NAFLD predisposition in children.

## Non-alcoholic fatty liver disease in childhood and adolescence

Moving forward to early childhood, there are currently little data on the status of the liver in children who are one to seven years old, but markers of MS can be seen in preschool-aged children. Prenatal and perinatal exposures of GDM and high birth weight are both established risk factors for MS in childhood ^36^. In addition, both high and low birth weight are correlated not only with NAFLD but also with increased risk of more advanced liver disease ^37^. Some evidence for NAFLD in preschool-aged children within high risk groups also exists. In a study mostly of Hispanic children in an obesity clinic, about one fourth of children starting at preschool age had elevated alanine aminotransferase (ALT) for age, and this proportion rose to nearly half by middle-school age ^38^.

NAFLD in most children is diagnosed in the peripubertal period. During puberty, IR transiently increases ^39, 40^, and children who are obese or have existing IR have a greater increase in IR during puberty, which may contribute to greater pathology in the liver ^41^. Histologically, children in puberty can display greater hepatic steatosis and more severe portal inflammation than adults, whereas lobular inflammation and ballooning appear milder in children compared with adults ^42^.

## Non-alcoholic fatty liver disease as a risk factor of cardiometabolic disease

The increase in IR and worsening of NAFLD can be somewhat of a “chicken or the egg” question. Few longitudinal studies exist in children to help answer this, but in a study of Japanese adults, after adjustment for other confounding factors, patients with fatty liver were more likely (hazard ratio (HR) 1.49) to become dysmetabolic compared with their counterparts without fatty liver ^43^. This supports the view of NAFLD as a driver of ongoing IR, visceral adiposity, and metabolic dysfunction ^15, 44^.

There is convincing evidence that NAFLD is an independent risk factor for cardiovascular disease events even after adjustment for conventional risk factors (odds ratio 4.12, 95% confidence interval (CI) 1.58 to 10.75, *P* = 0.004) ^45^. Significantly more patients with NAFLD had either coronary artery disease (7.5% versus 1.4%) or stroke (0.9% versus 0.2%) than non-NAFLD control patients ^46^. Data suggest that cardiac intima-media thickness (CIMT), an early predictor of atherosclerosis, and Framingham risk score significantly increase across quartiles of the fatty liver index (an accurate predictor of hepatic teatosis on ultrasonography) ^47, 48^. In addition, patients who developed hepatic steatosis showed an increase in CIMT whereas those without did not see a significant change ^47^. In regard to cardiac dysfunction risk, patients with NAFLD, particularly those with non-alcoholic steatohepatitis, have worsened right ventricular function compared with non-NAFLD counterparts ^49^. Coupled with type 2 diabetes, the risk for left ventricular dysfunction is also higher: patients with both T2DM and NAFLD have greater left ventricular diastolic dysfunction than those with T2DM alone ^50^.

There is also evidence that NAFLD may increase risk of T2DM ^44, 51^. One study found the prevalence of T2DM to be higher in patients with NAFLD when compared with their obese, non-NAFLD counterparts (6.5% versus <1%). Notably, girls with NAFLD were more likely to have T2DM than boys with NAFLD, despite an overall higher prevalence of NAFLD in boys ^52^. A longitudinal cohort study found that patients with only MS had a significantly higher risk of developing T2DM than those with NAFLD and no MS but that patients with both NAFLD and MS had the greatest risk of developing T2DM, almost four times greater than those with only NAFLD and two times greater than those with only MS. In addition, among those with MS, NAFLD significantly increased risk of T2DM (HR 1.93, 95% CI 1.38–2.71) ^53^. These authors hypothesized that the absence of an independent association between NAFLD and T2DM in those patients without MS may be due to differences in the severity of NAFLD ^53^. This theory is further supported by an independent study that found that NAFLD severity was associated with increased T2DM incidence even after controlling for MS and other predictors of T2DM ^54^.

## Conclusions

In summary, the intertwined stories of NAFLD and cardiometabolic dysregulation appear far more complex than previously hypothesized. In light of recent evidence suggesting that NAFLD may develop *in utero* and that NAFLD increases risk of both cardiovascular disease and T2DM, we contend that it is important to assess and treat NAFLD in childhood in order to prevent progression to these diseases. It is no longer possible to view NAFLD solely as a liver disease resulting from metabolic dysregulation; rather, it may be a causative factor in the spiral toward T2DM and cardiovascular events. The typical NAFLD clinical assessment is currently confined to liver pathology (steatosis, fibrosis, and screening for consequential cirrhosis and hepatocellular carcinoma), but this growing body of evidence emphasizes the importance of a broader patient assessment and demonstrates that NAFLD is a disease with systemic pathophysiologic consequences.

The practical applications of these conclusions are several. More knowledge is needed regarding hepatic fat in infants and young children and to understand whether hepatic steatosis disappears and reappears or is sustained from birth. This will require the development of a better and safer non-invasive measurement of fat in very young children. Furthermore, children in groups known to be at high risk for NAFLD should be studied longitudinally so that the onset of the liver disease can be better understood. This will be critical for developing a public health approach to prevent NAFLD and to test interventions that interrupt its course at the onset. Lastly, emerging evidence now suggests that, once children develop NAFLD, they are at high risk for the development of T2DM. This needs to be confirmed in a large-scale, long-term longitudinal study of pediatric NAFLD, and if it is confirmed, this population could be given priority for T2DM prevention programs.
